# Coexisting infectious diseases on admission as a risk factor for mechanical ventilation in patients with Guillain–Barré syndrome

**DOI:** 10.1016/j.je.2016.07.003

**Published:** 2017-03-07

**Authors:** Shinichiro Kobori, Tatsuhiko Kubo, Makoto Otani, Keiji Muramatsu, Yoshihisa Fujino, Hiroaki Adachi, Hiromasa Horiguchi, Kiyohide Fushimi, Shinya Matsuda

**Affiliations:** a Department of Preventive Medicine and Community Health, School of Medicine, University of Occupational and Environmental Health, Kitakyushu, Fukuoka, Japan; b Department of Occupational Health Data Science Center, University of Occupational and Environmental Health, Kitakyushu, Fukuoka, Japan; c Department of Neurology, School of Medicine, University of Occupational and Environmental Health, Kitakyushu, Fukuoka, Japan; d Department of Clinical Data Management and Research, Clinical Research Center, National Hospital Organization Headquarters, Tokyo, Japan; e Department of Health Policy and Informatics, Tokyo Medical and Dental University, Graduate School of Medicine, Tokyo, Japan

**Keywords:** Guillain–Barré syndrome, Coexisting infectious diseases, Mechanical ventilation

## Abstract

**Background:**

The aim of this study was to investigate patient characteristics on admission to hospital that increase the risk of subsequent mechanical ventilation (MV) use for patients with Guillain–Barré syndrome (GBS).

**Methods:**

We extracted data from the Japanese Diagnosis Procedure Combination (DPC) database for 4132 GBS patients admitted to hospital. Clinical characteristics of GBS patients with and without MV were compared. Multivariate logistic regression analyses were performed to estimate the odds ratios (ORs) and 95% confidence intervals (CIs) for the associations of requirement for MV with coexisting infectious diseases, after adjustment for potential confounding variables, age, sex, hospital type, and ambulance transportation.

**Results:**

In total, 281 patients required MV, and 493 patients had coexisting respiratory diseases on admission. After adjustment for covariates and stratification by coexisting respiratory diseases, multivariate logistic regression analysis revealed that coexisting cytomegaloviral (CMV) disease (OR 8.81; 95% CI, 2.34–33.1) and herpes simplex viral (HSV) infections (OR 4.83; 95% CI, 1.16–20.1) were significantly associated with the requirement for MV in the group without coexisting respiratory diseases.

**Conclusion:**

Our findings suggest that coexisting CMV and HSV infections on admission might be significantly associated with increased risk of respiratory failure in GBS patients.

## Highlights

• Subjects of this study were 4132 inpatients with Guillain–Barré syndrome.
• Data were derived from the Japanese Diagnosis Procedure Combination (DPC) database.
• Association between comorbidities and mechanical ventilation use was evaluated.
• Cytomegaloviral and herpes simplex viral infections were associated with ventilation.

## Introduction

Guillain–Barré syndrome (GBS) is an acute polyradiculoneuropathy and is considered to be the prototypical post-infectious autoimmunity.^1^ GBS is a heterogeneous syndrome with regard to severity of neurologic deficits and prognosis. Despite the availability of modern immunomodulatory treatments, such as plasma exchange and intravenous immunoglobulins, a number of GBS patients have fatal outcomes or suffer from long-term disability.^2^, ^3^

The main cause of death in patients with GBS is respiratory complications.^4^ Respiratory failure requiring ventilatory support occurs in approximately 20%–30% of GBS patients and is a life-threatening manifestation.^5^ The requirement for mechanical ventilation (MV) has been identified as a prominent prognostic indicator of poor outcome in GBS.^6^ Therefore, predicting patients who may require MV at an early stage of hospitalization is clinically important.

A number of studies have investigated potential predictors of requiring ventilatory support in patients with GBS. Among those studies, predictors of requiring MV in GBS have been divided into clinical, electrophysiological, and biological factors.^6^, ^7^

With regard to clinical predictors, disability grade on admission,^8^ rapid progressive motor weakness,^9^ bilateral facial weakness,^9^ bulbar dysfunction,^9^ dysautonomia,^10^ and a rapid decrease in vital capacity^10^, ^11^ have all been identified as risk factors. For electrophysiological factors, nerve conduction block^12^ and demyelinating form^12^ have been reported. In terms of biological factors, increased liver enzymes,^8^ increased plasma cortisol,^13^ anti-GQ1b antibodies,^14^ and positive cytomegaloviral (CMV) serology^15^ have all been identified in previous studies.

In about one-half to two-thirds of GBS patients, there is a history of preceding infectious disease, such as infection with *Campylobacter jejuni* (*C. jejuni*), CMV, Epstein–Barr virus, Mycoplasma pneumoniae, Haemophilus influenzae, or Herpes simplex virus.^2^, ^7^, ^16^, ^17^ Several case studies have also described GBS associated with serologically confirmed Herpes simplex viral (HSV) infection.^18^, ^19^

Preceding infections have a close relationship to the pathological mechanism of GBS.^7^ On the other hand, coexisting infectious diseases on hospital admission rather than preceding infections may be identified in some GBS patients. We are aware of no large-scale epidemiological studies that have evaluated the association between coexisting infectious diseases and subsequent MV in GBS patients. Therefore, we performed a large-scale epidemiological study to investigate the association between coexisting infectious diseases and requirement for MV in patients with GBS.

## Methods

### Database

Data were extracted from the Diagnosis Procedure Combination (DPC), the Japanese national case-mix system database constructed by the Ministry of Health, Labour and Welfare. As of 2010, the DPC database was used by 1648 acute hospitals in Japan. Of those, 980 hospitals participate in a DPC research group survey and provide their data for research purposes.

These data consist of approximately 4.93 million inpatients records, representing approximately 67% of all acute-care inpatient hospitalizations.^20^, ^21^

The DPC database contains each patient's discharge summary and administrative claims data, as well as date of birth, sex, dates of admission and discharge, and details of diagnosis. Disease names are recorded as main disease (the disease listed at the top of the disease list on the discharge summary), hospitalization disease (the primary disease that triggered admission), resource diseases (diseases that required use of hospital resources, mostly in terms of finance; a maximum of two diseases recorded), comorbidities (diseases that coexisted at admission; a maximum of four diseases recorded), and complications (diseases that occurred during admission; a maximum of four diseases recorded).^22^, ^23^, ^24^ These diagnoses are coded using the International Classification of Diseases and Injuries 10th Revision (ICD-10). DPC data also include the quantity and date of all care delivered on a daily basis. The research protocol of the study was approved by the ethics committee of medical care and research of Tokyo Medical and Dental University.

### Study setting

For this study, we extracted data from the DPC reported by the 866 hospitals (76 advanced treatment hospitals and 790 general hospitals) that participated in a DPC research survey from 2010 to 2012. From these data, 5375 patients were selected for whom both the hospitalization diseases and resource diseases were recorded as the ICD-10 code G610 (“Guillain–Barré syndrome”). After excluding 1132 patients with GBS affinity diseases (“Miller Fisher syndrome and Bickerstaff's Brainstem Encephalitis”), our dataset consisted of 4243 patients with GBS. We further excluded 111 patients who received MV on the first day of hospitalization, leaving 4132 patients for analysis. From the discharge summary data from the DPC database, data on age, sex, hospital type, use of ambulance transportation, and coexisting diseases on admission were extracted. Age was categorized into three groups: <40 years, 40–59 years, and ≥60 years. Hospital type was classified as advanced treatment hospital or general hospital.

### Study variables

Among coexisting diseases on admission, four known risk factors for MV were considered that could be identified using the DPC database: bulbar palsy (ICD-10 codes G12, R13),^9^ facial palsy (G51),^9^ autonomic dysfunction (G90, H57, I10, I49, I95, K56, K59, L74, N39, R00, R15, R32, R33, R39, R61),^10^ and diseases of the respiratory system (respiratory diseases) (J00-J99 except for malignant neoplasm, defined as C00-C97).^10^ To define cases with coexisting infectious diseases on admission, cases with ICD-10 codes A00-A99 or B00-B99 (certain infectious and parasitic diseases) were identified. To assess the association between specific infectious diseases (that is, ICD-10 codes to three digits) and subsequent MV, 13 coexisting infectious diseases with prevalence higher than 0.1% were selected. These diseases were infectious gastroenteritis and colitis, unspecified (ICD-10 code: A09); chronic viral hepatitis (B18); CMV disease (B25); bacterial infection of unspecified site (A49); HSV infections (B00); herpes zoster (B02); other viral diseases, not elsewhere classified (B33); other bacterial intestinal infections (A04); sepsis (A41); tuberculosis of the nervous system (A17); dermatophytosis (B35); viral meningitis (A87); and unspecified viral encephalitis (A86). Among these coexisting infectious diseases, four diseases were associated with requiring MV (P ≤ 0.2 using Pearson's chi-squared test) in the preliminary analysis and were selected as covariates for this study: CMV disease (B25), bacterial infection of unspecified site (A49), HSV infections (B00), and sepsis (A41).

### Statistical analysis

Clinical characteristics of GBS patients with and without MV were compared. Chi-squared tests were used to compare the proportions of categorical variables. Multivariate logistic regression models were applied to identify the association of coexisting infectious diseases on admission with requiring MV in GBS patients after adjustment for age, gender, hospital type, use of ambulance transportation, and the four known risk factors of MV found in previous studies. Respiratory diseases often develop into respiratory failure requiring MV. Therefore, multivariate logistic regression analysis was performed stratified by coexisting respiratory diseases (ICD-10 codes J00-J99). Further, univariate and multivariate logistic regression analyses were performed in the group of patients without coexisting respiratory diseases to evaluate the associations of the four coexisting infectious diseases with requiring MV. In the univariate and multivariate analysis, a P value < 0.05 was considered statistically significant. All statistical analysis was performed using Stata version 12.0 (Stata Corporation, College Station, TX, USA).

## Results

Table 1 summarizes the clinical characteristics of patients with GBS. Of the total of 4132 patients we identified, one group required MV (n = 281) and the other group did not require MV (n = 3851). The proportion of patients aged ≥60 years (49.5% vs. 33.5%, P < 0.001) and the proportion of patients using ambulance transportation (38.8% vs. 13.2%, P < 0.001) were significantly higher among those requiring MV than among those without MV. Among coexisting diseases on admission, the prevalence of respiratory diseases (44.1% vs. 9.6%, P < 0.001), bulbar palsy (12.8% vs. 4.8%, P < 0.001), CMV disease (1.8% vs. 0.4%, P = 0.002), bacterial infection of unspecified site (2.1% vs. 0.4%, P < 0.001), sepsis (1.4% vs. 0.1%, P < 0.001), and HSV infections (2.1% vs. 0.3%, P < 0.001) were significantly higher in patients with MV than in those without.

**Table 1:** Clinical characteristics and presentations of the patients with Guillain–Barré syndrome.

	Total	Without MV	With MV	P value^a^
Number of patients	4132	3851	281	
Age, years				
<40	40.2	41.2	26.3	<0.001
40–59	25.2	25.2	24.2	
≥60	34.6	33.5	49.5	
Male patients	58.7	58.3	64.1	0.058
Hospital type				
Advanced treatment hospitals	24.4	24.3	24.9	0.827
General hospitals	75.6	75.7	75.1	
Ambulance transportation	14.9	13.2	38.8	<0.001
Coexisting diseases on admission				
Respiratory diseases	11.9	9.6	44.1	<0.001
Bulbar palsy	5.3	4.8	12.8	<0.001
Facial palsy	2.4	2.5	0.7	0.054
Autonomic dysfunction	19.9	19.7	21.4	0.512
Certain infectious and parasitic diseases	5.5	5.2	9.6	0.002
Cytomegaloviral disease	0.5	0.4	1.8	0.002
Bacterial infection of unspecified site	0.5	0.4	2.1	<0.001
Herpes simplex viral infections	0.4	0.3	2.1	<0.001
Sepsis	0.2	0.1	1.4	<0.001

MV, mechanical ventilation.

Values are reported as %, unless otherwise noted.

a P values derived from Pearson's chi-squared test.

Table 2 represents multivariate logistic regression analysis of factors associated with requirement of MV. Regarding coexisting diseases on admission, certain infectious and parasitic diseases were significantly associated with increased odds of MV, after adjustment for potential confounding factors (odds ratio [OR] 1.85; 95% confidence interval [CI], 1.17–2.92). In addition, the association between coexisting respiratory diseases and subsequent MV showed strong statistical significance (OR 6.35; 95% CI, 4.85–8.33).

**Table 2:** Multivariate logistic regression analysis of characteristics associated with requirement for MV.

Independent variables	Odds ratio	95% CI	P value^a^
All patients (n = 4132)			
Age, years			
<40	1.00 (reference)		
40–59	1.56	(1.09, 2.23)	0.016
≥60	2.04	(1.47, 2.82)	<0.001
Sex			
Female	1.00 (reference)		
Male	0.76	(0.58, 0.99)	0.045
Hospital type			
General hospitals	1.00 (reference)		
Advanced treatment hospitals	1.17	(0.86, 1.58)	0.310
Ambulance transportation			
Not used	1.00 (reference)		
Used	3.15	(2.39, 4.17)	<0.001
Coexisting diseases on admission			
Without respiratory diseases	1.00 (reference)		
With respiratory diseases	6.35	(4.85, 8.33)	<0.001
Without bulbar palsy	1.00 (reference)		
With bulbar palsy	1.96	(1.28, 2.98)	0.002
Without facial palsy	1.00 (reference)		
With facial palsy	0.36	(0.09, 1.48)	0.156
Without autonomic dysfunction	1.00 (reference)		
With autonomic dysfunction	0.94	(0.68, 1.30)	0.708
Without certain infectious and parasitic diseases	1.00 (reference)		
With certain infectious and parasitic diseases	1.85	(1.17, 2.92)	0.009

CI, confidence interval; MV, mechanical ventilation.

a Multivariate logistic regression analysis adjusted for age, sex, hospital type, ambulance use, and coexisting diseases on admission.

Table 3 demonstrates multivariate logistic regression analysis of factors associated with need for MV stratified by coexisting respiratory diseases (ICD-10 codes J00-J99) on admission. In the group with coexisting respiratory diseases, coexisting infectious diseases were not significantly associated with subsequent MV (OR 1.49; 95% CI, 0.70–3.17). In the group without coexisting respiratory diseases, coexisting infectious diseases were significantly related to the need for MV (OR 2.03; 95% CI, 1.14–3.60).

**Table 3:** Multivariate logistic regression analysis of characteristics associated with requirement for MV stratified by coexisting respiratory diseases.

Independent variables	With coexisting respiratory diseases			Without coexisting respiratory diseases		
	Odds ratio	95% CI	P value^a^	Odds ratio	95% CI	P value^b^
All patients		n = 493			n = 3639	
Age, years						
<40	1.00 (reference)			1.00 (reference)		
40–59	1.82	(1.00, 3.32)	0.051	1.44	(0.92, 2.26)	0.114
≥60	2.21	(1.31, 3.74)	0.003	1.91	(1.27, 2.89)	0.002
Sex						
Female	1.00 (reference)			1.00 (reference)		
Male	0.66	(0.42, 1.02)	0.064	0.85	(0.60, 1.19)	0.335
Hospital type						
General hospitals	1.00 (reference)			1.00 (reference)		
Advanced treatment hospitals	1.39	(0.84, 2.31)	0.200	1.11	(0.76, 1.62)	0.596
Ambulance transportation						
Not used	1.00 (reference)			1.00 (reference)		
Used	2.94	(1.85, 4.65)	<0.001	3.32	(2.34, 4.71)	<0.001
Coexisting diseases on admission						
Without bulbar palsy	1.00 (reference)			1.00 (reference)		
With bulbar palsy	1.35	(0.69, 2.62)	0.383	2.46	(1.45, 4.17)	0.001
Without facial palsy	1.00 (reference)			1.00 (reference)		
With facial palsy	(omitted)	–	–	0.44	(0.11, 1.83)	0.260
Without autonomic dysfunction	1.00 (reference)			1.00 (reference)		
With autonomic dysfunction	0.74	(0.41, 1.32)	0.304	1.05	(0.71, 1.57)	0.795
Without certain infectious and parasitic diseases	1.00 (reference)			1.00 (reference)		
With certain infectious and parasitic diseases	1.49	(0.70, 3.17)	0.300	2.03	(1.14, 3.60)	0.015

CI, confidence interval; MV, mechanical ventilation.

a Multivariate logistic regression analysis in the group with coexisting respiratory diseases adjusted for age, sex, hospital type, ambulance use, and coexisting diseases on admission.

b Multivariate logistic regression analysis in the group without coexisting respiratory diseases adjusted for age, sex, hospital type, ambulance use, and coexisting diseases on admission.

Table 4 shows univariate and multivariate logistic regression analysis of factors associated with the requirement of MV in the group without coexisting respiratory diseases on admission. In univariate logistic regression analysis, CMV disease (OR 6.98; 95% CI, 2.25–21.6), bacterial infection of unspecified site (OR 8.25; 95% CI, 2.60–26.2), HSV infections (OR 6.76; 95% CI, 1.84–24.8), and sepsis (OR 11.2; 95% CI, 2.04–61.7) were significantly associated with subsequent need for MV. In multivariate logistic regression analysis, CMV disease (OR 8.81; 95% CI, 2.34–33.1) and HSV infections (OR 4.83; 95% CI, 1.16–20.1) were significantly associated with increased odds of MV. Similarly, age ≥60 years (OR 2.03; 95% CI, 1.33–3.09), ambulance transportation (OR 3.27; 95% CI, 2.30–4.66), and bulbar palsy (OR 2.47; 95% CI, 1.45–4.21) were significantly associated with increased odds of MV (Hosmer–Lemeshow goodness of fit test P = 0.702).

**Table 4:** Univariate and Multivariate logistic regression analysis of characteristics associated with requirement for MV in the group without coexisting respiratory diseases.

Independent variables	Univariate analysis			Multivariate analysis		
	Odds ratio	95% CI	P value^a^	Odds ratio	95% CI	P value^b^
All patients (n = 3639)						
Age, years						
<40	1.00 (reference)			1.00 (reference)		
40–59	1.53	(0.98, 2.38)	0.059	1.52	(0.96, 2.40)	0.075
≥60	2.21	(1.50, 3.25)	<0.001	2.03	(1.33, 3.09)	0.001
Sex						
Female	1.00 (reference)			1.00 (reference)		
Male	0.81	(0.58, 1.12)	0.202	0.86	(0.61, 1.21)	0.400
Hospital type						
General hospitals	1.00 (reference)			1.00 (reference)		
Advanced treatment hospitals	0.97	(0.67, 1.41)	0.875	1.11	(0.76, 1.63)	0.584
Ambulance transportation						
Not used	1.00 (reference)			1.00 (reference)		
Used	3.77	(2.67, 5.31)	<0.001	3.27	(2.30, 4.66)	<0.001
Coexisting diseases on admission						
Without bulbar palsy	1.00 (reference)			1.00 (reference)		
With bulbar palsy	3.04	(1.83, 5.04)	<0.001	2.47	(1.45, 4.21)	0.001
Without facial palsy	1.00 (reference)			1.00 (reference)		
With facial palsy	0.47	(0.11, 1.93)	0.294	0.32	(0.07, 1.43)	0.135
Without autonomic dysfunction	1.00 (reference)			1.00 (reference)		
With autonomic dysfunction	1.28	(0.88, 1.86)	0.199	1.05	(0.71, 1.57)	0.797
Certain infectious and parasitic diseases						
Without cytomegaloviral disease	1.00 (reference)			1.00 (reference)		
With cytomegaloviral disease	6.98	(2.25, 21.6)	0.001	8.81	(2.34, 33.1)	0.001
Without bacterial infection of unspecified site	1.00 (reference)			1.00 (reference)		
With bacterial infection of unspecified site	8.25	(2.60, 26.2)	<0.001	3.81	(0.86, 16.9)	0.078
Without herpes simplex viral infections	1.00 (reference)			1.00 (reference)		
With herpes simplex viral infections	6.76	(1.84, 24.8)	0.004	4.83	(1.16, 20.1)	0.030
Without sepsis	1.00 (reference)			1.00 (reference)		
With sepsis	11.2	(2.04, 61.7)	0.005	7.25	(0.91, 57.5)	0.061

CI, confidence interval; MV, mechanical ventilation.

a Univariate logistic regression analysis of following characteristics, age, sex, hospital type, ambulance use, and coexisting diseases on admission.

b Multivariate logistic regression analysis of characteristics adjusted for age, sex, hospital type, ambulance use, and coexisting diseases on admission.

## Discussion

In this study, multivariate analysis demonstrated that coexisting infectious diseases and coexisting respiratory diseases on admission were significantly associated with the requirement for subsequent MV. A second multivariate analysis stratified according to coexisting respiratory diseases revealed that coexisting infectious diseases were significantly associated with the requirement for MV in the group without coexisting respiratory diseases. A third multivariate analysis suggested that coexisting CMV and HSV infection were significantly associated with the need for ventilatory support in GBS patients, after adjustment for other comorbidities and other factors.

Several studies demonstrate age as a predictor of poor outcome in GBS.^5^, ^6^ GBS patients who need MV often have poorer prognosis than those who do not need MV.^6^ Therefore, older GBS patients may have a higher risk of subsequent MV. GBS patients who need for ambulance transportation may have higher disease severity than those who do not need ambulance transportation. Our present findings about the association between coexisting bulbar palsy on admission in GBS patients and subsequent MV are consistent with past studies.^9^

In addition, the findings regarding the association between coexisting CMV infections and the requirement for MV are consistent with some previous studies.^15^ CMV infections are the second-most prevalent preceding infection for GBS patients next to *C. jejuni*, occurring in 11%–15% of patients.^17^ CMV-related GBS tends to follow a severe course indicated by the high frequency of respiratory insufficiency.^15^ This association may be explained by the observation that CMV-related GBS patients tend to have more severe demyelination than the other patients. Demyelinating lesions are often located in nerves involved in respiration.^15^ Furthermore, because CMV itself can lead to pneumonia, patients with CMV-related GBS may develop respiratory insufficiency or require ventilator assistance.

Our study has provided evidence that coexisting HSV infections on admission might be significantly associated with subsequent MV in GBS patients. In previous studies, HSV-related GBS patients developed respiratory muscle weakness and required ventilatory assistance.^25^, ^26^ The following mechanisms may explain the relationship between HSV-related GBS and requirement for MV. First, HSV infections are often complicated by diaphragmatic weakness, which may lead to respiratory failure or the requirement for respirator management in patients with GBS.^27^, ^28^ Diaphragm weakness has been found to be the main mechanism of respiratory failure in patients with GBS.^27^ Demyelination of the phrenic nerve has been mentioned as a cause of the diaphragmatic paralysis.^28^ The etiologies of diaphragmatic paralysis can be post-surgical (e.g., phrenic nerve transection or damage secondary to retraction or electrocautery), neoplastic (e.g., direct phrenic nerve invasion or metastatic involvement), and neuromuscular (e.g., sequelae of myelitis, encephalitis, polio, diphtheria, or multiple sclerosis). HSV infections have been considered one of the neuromuscular etiologies of diaphragmatic paralysis.^29^

Second, HSV infections in this study include HSV encephalitis (HSE). HSE is one of the most severe infections of the central nervous system and manifests throughout the year in patients of all ages.^30^ Patients with HSE often develop acute consciousness impairment. We assumed that an alteration of consciousness in HSE patients increased the risk of airway obstruction or aspiration pneumonia and subsequent respiratory failure.^30^

HSV Immunoglobulin M (IgM) and HSV Immunoglobulin G (IgG) antibody measurement using polymerase chain reaction (PCR) methods have been used as diagnostic procedure for HSV.^31^ However, HSV IgM and HSV IgG antibody measurements may be unsuitable for early diagnosis on hospital admission, as PCR methods often give false-negative results during the early stages of hospitalization.^31^, ^32^ Furthermore, HSV infections only account for approximately 1% of preceding infections in GBS.^33^ For these reasons, evidence of the association with HSV infections and the requirement for MV has been considered to be scarce in previous studies.^33^, ^34^

The strengths of this study are derived from the DPC database. First, the large-scale database enables the evaluation of the association between HSV infections and subsequent MV, although HSV infections have been relatively rare as a preceding infection in GBS patients. Second, we obtained data for coexisting diseases on admission from the DPC database. Some HSV infections might be relatively easily diagnosed through physical examination, including skin examination at an early stage of hospitalization. In a prospective study of new infections with HSV type 1 and type 2, an acquisition of HSV infection was defined by the demonstration of either HSV type 1 or HSV type 2 seroconversion, a positive culture for HSV, or both.^34^ Of the new HSV type 2 infections, 37% were symptomatic, 82% of which were given a correct clinical diagnosis at presentation.^34^ Detection of the signs or symptoms of HSV infections at an early stage of hospitalization in GBS patients may be useful to estimate the risk of the need for ventilatory support.^35^ Considering that 63% of all HSV type 2 infections were asymptomatic,^34^ the risk of requiring MV may be underestimated. Technological developments to improve examination may help to detect GBS patients who have a high risk of requiring MV during hospitalization.

Several limitations are inherent to this study. First, GBS patients who were intubated or mechanically ventilated on the first day of hospitalization were excluded from the analysis. Therefore, we could not evaluate associations for patients whose condition exacerbated rapidly enough to lead to MV on the first day of admission. However, we excluded patients who were taken by ambulance with MV on admission. Second, we could not evaluate the history of treatments for the preceding infections in GBS patients because the DPC database includes only hospitalization data. Most antecedent infectious diseases associated with GBS are likely to have been cleared prior to admission to hospital.^36^ Finally, our study may include misclassification error owing to the use of ICD-10 codes corresponding to coexisting infectious diseases that have not been correctly applied. Consequently, the risk of GBS patients requiring MV may be underestimated. However, the influence of coexisting infectious diseases on the risk of MV in GBS cannot be explained wholly by misclassification error.

Despite these limitations, our study demonstrates that coexisting infectious diseases on admission may increase the risk of respiratory failure for GBS patients. Detecting clinical symptoms or signs that suggest the presence of coexisting infectious diseases may help to predict the short-term prognosis in these GBS patients. Screening GBS patients at high risk of respiratory failure at an early stage of hospitalization may help decision-making for admission to intensive care or preparation of a respirator prior to admission. Even if symptoms of GBS patients progress rapidly, an increase in survival rate may be seen. We believe that a fundamental evaluation of patients with GBS, based on careful interview and physical examination during the early stage of admission, is essential for improved prognosis.

In conclusion, our results suggest that coexisting infectious diseases on admission, primarily CMV and HSV infections, could represent potential risk factors of MV in GBS patients. Detecting the presence of coexisting infectious diseases on admission in GBS patients may have crucial implication for predicting the short-term clinical course of GBS and achieving better clinical outcomes.

## Conflicts of interest

None declared.
